# A genome-wide association study coupled with machine learning approaches to identify influential demographic and genomic factors underlying Parkinson’s disease

**DOI:** 10.3389/fgene.2023.1230579

**Published:** 2023-09-29

**Authors:** Md Asad Rahman, Jinling Liu

**Affiliations:** ^1^ Department of Engineering Management and Systems Engineering, Missouri University of Science and Technology, Rolla, MO, United States; ^2^ Department of Biological Sciences, Missouri University of Science and Technology, Rolla, MO, United States

**Keywords:** Parkonson’s disease, genome-wide association studies, machine learning, prediction model, feature importance analysis

## Abstract

**Background:** Despite the recent success of genome-wide association studies (GWAS) in identifying 90 independent risk loci for Parkinson’s disease (PD), the genomic underpinning of PD is still largely unknown. At the same time, accurate and reliable predictive models utilizing genomic or demographic features are desired in the clinic for predicting the risk of Parkinson’s disease.

**Methods:** To identify influential demographic and genomic factors associated with PD and to further develop predictive models, we utilized demographic data, incorporating 200 variables across 33,473 participants, along with genomic data involving 447,089 SNPs across 8,840 samples, both derived from the Fox Insight online study. We first applied correlation and GWAS analyses to find the top demographic and genomic factors associated with PD, respectively. We further developed and compared a variety of machine learning (ML) models for predicting PD. From the developed ML models, we performed feature importance analysis to reveal the predictability of each demographic or the genomic input feature for PD. Finally, we performed gene set enrichment analysis on our GWAS results to identify PD-associated pathways.

**Results:** In our study, we identified both novel and well-known demographic and genetic factors (along with the enriched pathways) related to PD. In addition, we developed predictive models that performed robustly, with AUC = 0.89 for demographic data and AUC = 0.74 for genomic data. Our GWAS analysis identified several novel and significant variants and gene loci, including three intron variants in *LMNA* (*p*-values smaller than 4.0e-21) and one missense variant in *SEMA4A* (p-value = 1.11e-26). Our feature importance analysis from the PD-predictive ML models highlighted some significant and novel variants from our GWAS analysis (e.g., the intron variant rs1749409 in the *RIT1* gene) and helped identify potentially causative variants that were missed by GWAS, such as rs11264300, a missense variant in the gene *DCST1*, and rs11584630, an intron variant in the gene *KCNN3*.

**Conclusion:** In summary, by combining a GWAS with advanced machine learning models, we identified both known and novel demographic and genomic factors as well as built well-performing ML models for predicting Parkinson’s disease.

## 1 Introduction

Parkinson’s disease (PD) is a complex neurodegenerative disorder often linked to aging (Dauer and Przedborski, 2003). Symptoms of Parkinson’s can be broadly divided into motor and non-motor categories (Sveinbjornsdottir, 2016). Primary motor symptoms of PD include bradykinesia, tremor, and rigidity (Xia and Mao, 2012). Other manifestations involve gait disturbances, impaired handwriting, grip force (related to the strength and control of hand grasping), and speech deficits (Moustafa et al., 2016). In PD, non-motor symptoms are categorized into sensory symptoms, neuropsychiatric dysfunctions, autonomic dysfunction, and sleep disorders (Poewe, 2008). Among these, sensory symptoms may include olfactory dysfunction, abnormal sensations, and pain. Neuropsychiatric dysfunctions can encompass mood disorders, frontal executive dysfunction, apathy, and anhedonia. Autonomic dysfunction might present symptoms like orthostatic hypotension, urogenital dysfunction, and constipation. Lastly, sleep disturbances can involve sleep fragmentation, insomnia, and rapid eye movement sleep behavior disorder.

In addition to age, environmental, and genomic factors also contribute to the development of PD (Noyce et al., 2012; Kieburtz and Wunderle, 2013; Blauwendraat et al., 2020). Specific environmental factors, such as exposure to pesticides and smoking, are associated with an increased risk of PD; conversely, caffeine intake is also linked to a decreased risk of PD. Advancements in high-throughput technologies have enabled genome-wide association studies (GWAS) to detect significant associations between genomic variants and various diseases, including PD. Following the identification of the first PD GWAS loci in 2009, 90 distinct risk loci have been discovered thus far (Nalls et al., 2014; Chang et al., 2017; Visscher et al., 2017; Nalls et al., 2019). Despite these current successes, many more significant variants are yet to be discovered to explain the genomic heritability of PD.

The increasing number of risk loci identified by GWAS has helped improve PD prediction and intervention (Chairta et al., 2021; Kim et al., 2021; Salas-Leal et al., 2021; Zheng et al., 2021; Dehestani et al., 2022). In prior studies, polygenic risk scores (PRS) were used to predict the risk of PD. These scores captured the cumulative effect of various PD genetic variants. Typically, the effectiveness of this PRS method for PD prediction was indicated by the value of an area under the receiver operating characteristics curve (AUC) ranging from 0.61 to 0.69 (Nalls et al., 2019; Chairta et al., 2021; Kim et al., 2021; Salas-Leal et al., 2021; Zheng et al., 2021; Dehestani et al., 2022). The prediction performance needs further improvement for the genomic prediction of PD to have clinical use. In addition, the PRS model lacks knowledge of specific variants’ involvement and their magnitude of impact for predicting PD risks. At the same time, many other studies have explored the utility of the existing demographic and clinical data (e.g., motor and non-motor symptoms) for predicting PD risks (Nielsen et al., 2017; Zham et al., 2017; Shah et al., 2018; Senturk, 2020). The application of advanced machine learning (ML) models with a combined feature space including genomic, demographic, and clinical data may further improve the accuracy of PD prediction.

In this study, the main purpose was to examine the key factors influencing PD by utilizing a large dataset containing demographic, clinical, and genetic data from the Fox Insight online study (Smolensky et al., 2020). This aim contained three key components: examining demographic and clinical variables through correlation and feature importance analyses, studying genomic factors using GWAS and feature importance analysis, and developing machine learning models for PD prediction. To find demographic and clinical variables associated with PD, we conducted a comprehensive analysis involving correlation assessment and feature importance analyses. To identify the potential genomic causes, we initially applied GWAS to the newly released genetic data by the Fox Insight study to search for novel and significant genomic variants for PD. Subsequently, we selected the top GWAS variants as input features to develop ML models for PD prediction; we applied and compared the performance of four popular ML models: artificial neural networks (ANNs), random forest (RF), support vector machine (SVM), and logistic regression (LR) (Svozil et al., 1997; Liaw and Wiener, 2002; Noble, 2006; Sperandei, 2014). Our strategy involved constructing three different kinds of ML predictive models: a demographic model (using demographic and clinical data only), a genetic model (using genetic data only), and a combined model (using both genetic and demographic/clinical data). Furthermore, we investigated and identified the most predictive demographic variables and genomic variants using two different feature importance methods: expected gradients applied to ANNs and feature importance score given by RF (Louppe et al., 2013). Lastly, we performed GWAS-based gene set enrichment analysis (GSEA) using our GWAS results and identified novel and known PD pathways.

## 2 Materials and methods

### 2.1 Data and data preprocessing

In Fox Insight, participants were genotyped on the V3, V4, and V5 platforms. The V5 platform consisted of a customized Illumina Infinium Global Screening Array containing approximately 690,000 SNPs. Roughly 80.4% of participants were genotyped on this platform. We used the V5 platform of Fox Insight Genetic Data. We applied the following functions from plink for further filtration and quality control to each chromosome: --mind 0.05 --geno 0.03 --maf 0.01 –hwe 1e-6. We then imputed the missing SNP values (0.63% missing values) with the most frequent value for that particular SNP across the entire dataset. Dominant coding was then performed, and thus, the final SNP values are 0 or 1. After combining all 22 chromosomes, we obtained a total of 447,089 SNPs and 8,840 Samples. Phenotype data included the ‘CurrPDDiag’ variable which was downloaded using the Fox DEN tool. Participants who answered the registration question “Do you currently have a diagnosis of Parkinson’s disease, or Parkinsonism, by a physician or other healthcare professional?” were represented by the ‘CurrPDDiag’ variable.

We also processed the demographic and clinical data (one-time questionnaires and routine longitudinal assessments data, referred as demographic data later for convenience) that were also downloaded from the Fox DEN tool. The routine longitudinal assessment dataset was generated through routine longitudinal health and medical questionnaires, and the one-time questionnaire dataset was about environmental exposure and healthcare preferences (Smolensky et al., 2020). Initially, all downloaded demographic data from the Fox DEN tool had 53k samples and 5,877 demographic variables. We kept the demographic variables shared between PD and non-PD individuals. We used the most recent record for each of these variables. Furthermore, we selected the subjects who also have genotype data available (∼8k samples). Among the subjects with demographic and genetic data, we identified and removed demographic variables with missingness >5% in these individuals; we also removed variables that leak the PD information unsuitable for prediction, which left us 200 demographic variables. We further removed from the ∼53k samples the individuals who have missingness >5% in these selected 200 variables, which left us 33,473 samples and 200 variables.

### 2.2 Genome-wide association studies

GWAS is the standard approach for identifying the significant variants associated with traits at the population level. GWAS was performed using logistic regression adjusting for age (age at the onset for cases and age of last reported for controls), sex, and 10 principal components. We performed GWAS using R software (http://www.r-project.org/). The *p*-values from GWAS were used to evaluate whether corresponding SNPs were genome-wide significant or not. We used the Bonferroni correction method for selecting a threshold *p*-value of genome-wide significance (Kaler and Purcell, 2019).

### 2.3 Feature selection and machine learning model development

We divided the whole genetic dataset into an 80% training set, a 10% validation set, and a 10% test set containing 7,072, 884, and 884 subjects, respectively. We used the training set for feature selections through GWAS analysis and for training the model. The top SNPs with the lowest *p*-values from GWAS analysis were selected as potentially informative input features for ML models to predict the PD status. We reserved an intact validation set for tuning hyperparameters and finding the best ML model and an intact test set for the performance evaluation of the final ML model. Fox Insight studies had a highly unbalanced case-control ratio of around 30:1, so we applied random oversampling for the minority class in the training set to make a 2:1 (case-control) ratio for training the ML models. The oversampling method was not applied to GWAS analyses that were performed using the original data. The random oversampling method was not used in the validation or the test set either; thus, these sets consisted of actual data from Fox Insight to avoid both overfitting and reflect the actual performance. Both the validation and test set were unseen during GWAS analyses and training of the models to avoid potential information leakage. We used artificial neural networks (ANNs), random forest (RF), support vector machine (SVM), and logistic regression (LR) to predict the risk status of PD. The ANN was implemented using Keras while RF, SVM, and LR were implemented by using scikit-learn packages (Pedregosa et al., 2011; Chollet, 2015).

We also developed RF and ANN models to predict PD using demographic data. From the aforementioned processed demographic data containing 33,473 samples and 200 demographic variables, we performed a stratified random split to produce an 80% training set (*n* = 26,765), a 10% (*n* = 3,354) validation set, and a 10% test set (*n* = 3,354). Within the training data, we employed multiple correlation techniques on a total of 200 variables to determine the most relevant features for our analysis. We applied the Matthews correlation coefficient to 188 binary variables, Cramer’s V to 11 categorical variables with more than two discrete values, and the point-biserial correlation to one continuous variable (Kornbrot, 2014; Akoglu, 2018; Chicco et al., 2021). A threshold of 0.01 allowed us to identify a total of 139 variables that met our inclusion criteria. We further used the training set to tune the hyperparameters for both ANN and RF models based on the prediction performance on the validation set; we then used both the training and validation sets to train the final model that was used to predict the unseen test dataset. Furthermore, we developed a combined prediction model using both demographic and genetic features from subjects who have both demographic and genetic data. To comprehensively evaluate the prediction performance of the developed ML models in an unseen test dataset, we examined multiple metrics including the area under the ROC curve (AUC), precision, recall, and the F1-score (the harmonic mean of precision and recall).

### 2.4 Interpretation using feature importance and expected gradients

Mean decrease impurity (MDI) feature importance score is one of the methods used in the RF model to measure the relative importance of each input feature (Louppe et al., 2013). We applied “feature_importance_” (FI) to the RF model for identifying top features, later referred to as “RF FI.”

Shapley value is one of the most known methods that can interpret complex ML models and show the most impactful features. We applied the expected gradient (EG) method to the ANN model, later referred to as ANN EG. EG, an extension of the integrated gradient method, has a strong theoretical justification for finding the most important and contributing input features (e.g., SNPs and demographic factors) for the model’s prediction by approximating the Shapley value (Erion et al., 2021). It has a set of axioms: implementation invariance, sensitivity, completeness, linearity, and symmetry preserving (Erion et al., 2021). We implemented EG using the SHAP (SHapley Additive exPlanations) Python package. The SHAP value from EG indicates the overall impact on predictions as well as the directionality of that impact indicated by positive or negative values. The mean absolute SHAP value for each feature across all of the data emphasizes the significant features for prediction, regardless of their directionality.

### 2.5 Gene set enrichment analysis

GSEA was used to identify KEGG pathways significantly associated with PD. We used the minimum *p*-value among all SNPs near a gene to represent the significance of that gene (Wang et al., 2007). Later, GSEA software was used to calculate the enrichment score (ES) and false discovery rate (FDR) q-value. The ES is the highest departure from zero that is observed during the walk, and FDR is utilized to control the rate of false positive findings in hypothesis testing, especially in multiple testing scenarios. We used ‘GSEAPreranked,’ a module of the GSEA software, and provided it a list of genes that were ordered based on −log10 (*p*-value). For multiple hypothesis testing corrections, 1,000 random permutations were carried out by gene set. In order to generate a normalized enrichment score (NES), the ES for each gene set was normalized so that it accurately reflects the size of each gene set, and FDR was further calculated corresponding to each NES.

## 3 Results

### 3.1 Predictive ML models for PD developed from demographic data

We developed a set of ML models to predict the PD status from demographic data. As was described in Section 2, we obtained a short list of 139 demographic variables from the initial 5,700 variables in 33,473 subjects. We developed from the training (80%; *n* = 26,765) and validation sets (10%; *n* = 3,354) both an RF model and an ANN model; the ANN model was trained using the SGD algorithm (batch size: 8, sigmoid activation functions, learning rate: 0.01, and 16 neurons in one hidden layer). The prediction performance of the final model was evaluated in the unseen test dataset (10%; *n* = 3,354) using multiple metrics including AUC, precision, recall, and F1-score (Table 1). With this relatively large demographic dataset, both the RF and the ANN showed very good performance in predicting the PD status from the 139 demographic variables: both models achieved a high AUC of 0.89; for precision, the RF had 0.82 while the ANN had 0.81; for recall, the RF had 0.77 while the ANN had 0.79; and for F1-score, the RF had 0.79 while the ANN had 0.80.

**TABLE 1:** Performance metrics of the demographic ML models for predicting PD.

ML model	AUC	Precision	Recall	F1-score
RF	0.89	0.82	0.77	0.79
ANN	0.89	0.81	0.79	0.80

**FIGURE 1:**
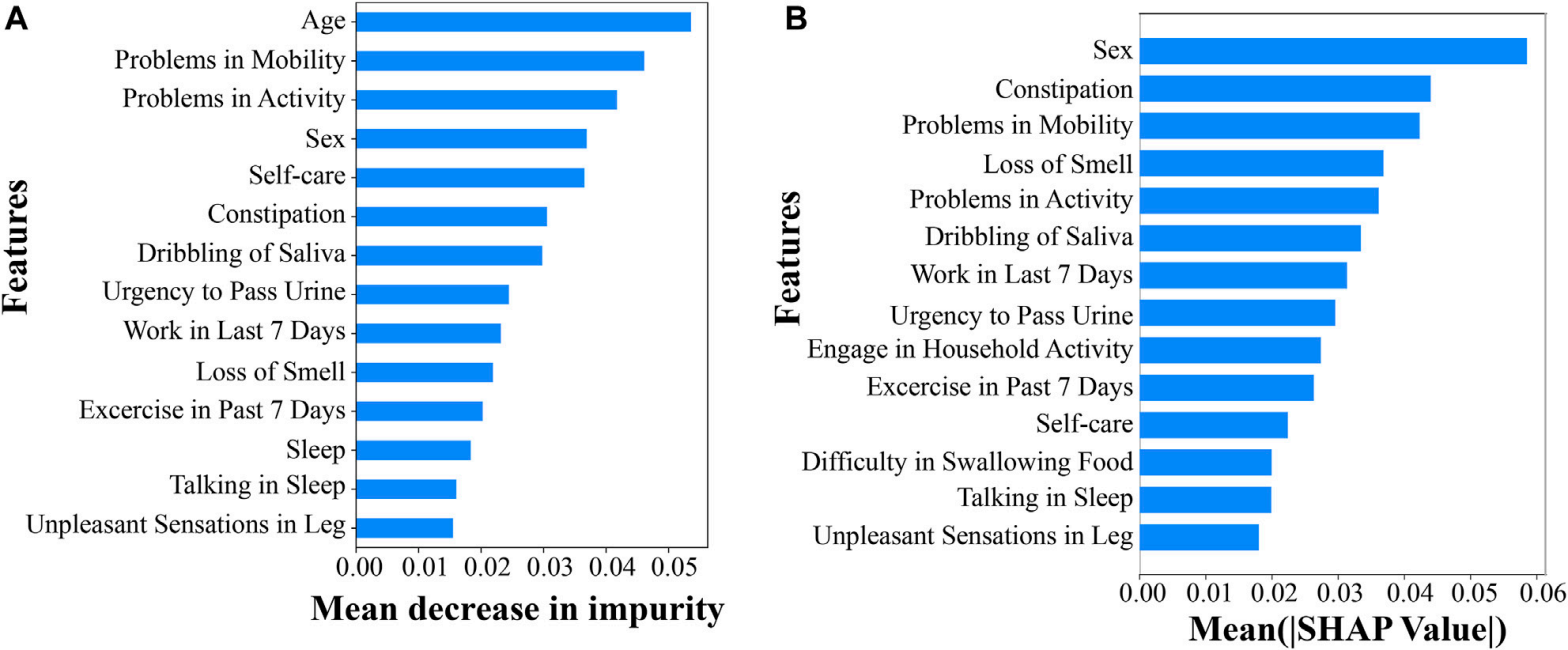
Top predictive demographic or clinical variables for PD. **(A)** Top 14 demographic variables by RF feature importance scores and **(B)** top 14 demographic variables by the EG method.

To understand the predictive performance of each of the 139 demographic variables, we acquired the feature importance score from the RF demographic model as well as performed EG analysis for the ANN demographic model. From the RF model, we listed the top 14 demographic variables that lead to the highest mean decrease in impurity and, thus, the most important features in predicting PD ranked by the RF model (Figure 1A). Similarly, from the EG analysis for the ANN model, we identified the top 14 demographic variables that show higher mean absolute SHAP values and, thus, more predictive power in predicting PD in the ANN model (Figure 1B). Interestingly, the feature importance and predictability ranked by these two methods from analyzing two different ML models were highly consistent for the top 14 demographic variables, with 12 being overlapped with each other (Figures 1A, B). The three variables of “sex,” “problems in mobility,” and “problems in activity” were ranked within the top 5 predictive variables for PD by both RF FI and ANN EG. Other top variables included constipation, loss of smell, dribbling of saliva, work in last 7 days, urgency to pass urine, engage in household activity, exercise in past 7 days, self-care, difficulty swallowing food or drink, talking or moving about in sleep, and unpleasant sensations in legs (Figure 1A). Multiple previous studies together identified most of these or very similar variables as significant variables associated with PD (Nielsen et al., 2017; Prashanth and Roy, 2018; Lo et al., 2019; Shah et al., 2020; Yu et al., 2022); this comprehensive list of top demographic/clinical variables identified in our study, based on their capability in predicting PD, adds further support to the influence of these factors in PD prediction. One of the top variables that has not been studied much is “unpleasant sensations in legs,” which is ranked 14th among all the 139 demographic variables by both RF FI and ANN EG. Yet, the exact question in the online survey for collecting information for this variable is “have you experienced unpleasant sensations in your legs at night or while resting, and a feeling that you need to move in the last month?” and this question is generally used as the first of the three questions in identifying restless legs syndrome (RLS) that is associated with PD (Wong et al., 2014). This suggested that the variable of “unpleasant sensation in legs” could be used a predictor for PD even before people were diagnosed having RLS.

All the non-overlapping variables between the top 14 lists of the two models were ranked very similarly by RF FI and ANN EG, except for “age.” Specifically, ‘sleep’, ‘engagement in household activity’, and ‘difficulty in swallowing food’ were ranked 12th, 16th, and 17th, respectively, by RF FI, while they were ranked 15th, 9th, and 12th, respectively, by ANN EG; “age” was ranked first by RF FI and 93rd by ANN EG. The much lower ranking of “age” by the EG method for the ANN model is likely because the ANN model does not handle a mixture of categorical and continuous variables well, with the EG method being biased for the continuous variable of age.

### 3.2 GWAS in the discovery (training) dataset, identifying both novel and well-known variants and genes of significance

**FIGURE 2:**
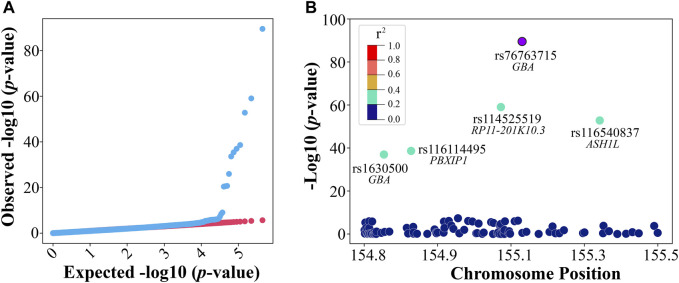
GWAS in discovery. **(A)** The quantile–quantile (QQ) plot was observed against expected *p*-values from the genome-wide association analysis and **(B)** a regional association plot of the rs76763715 locus.

To explore significant genomic variants, we applied GWAS to the preprocessed discovery (training) dataset that includes 6,868 PD cases and 204 controls with 447,089 SNPs. Males comprised 55% (*n* = 3,885) of the discovery dataset, and the rest are females (*n* = 3,187). A quantile–quantile plot was constructed for all variants by comparing expected vs. observed genome-wide *p*-values as a quality control for the GWAS analysis (Figure 2A). For the GWAS analysis, if considering 0.05/447,089 = 1.12e-07 as the significance level for *p*-value after the Bonferroni correction (Ranstam, 2016), 14 SNPs reached such significance (Supplementary Table S1). Among these 14 SNPs, two variants, rs76763715 (alias, i4000415), a missense variant in *GBA*, and rs1630500, an intergenic variant in *GBA*, as well as three gene loci, *GBA*, *ARHGEF2*, and *LMNA*, were previously reported for PD association (Redenšek et al., 2017; Ferrari et al., 2018; Oyston et al., 2018). The well-known variant of rs76763715 showed the most significant association with PD in our GWAS analysis. The regional association plot revealed that within a ± 400-kb window, several significant SNPs (green) on chromosome 1 had a moderate level of coefficient of determination (r_squared ≥ 0.2) with rs76763715 (purple) (Figure 2B).

In addition to the previously reported variants or gene loci, our GWAS analysis also identified novel and significant variants or gene loci that could have a potential influence on PD. Three novel intron variants in the PD-associated *LMNA* gene loci were among the 14 significant SNPs with *p*-values smaller than 4.0e-21 (Supplementary Table S1); this finding further supported previous reports on the involvement of *LMNA* in PD. A novel missense variant in the *SEMA4A* loci was identified with a very low *p*-value of 1.11e-26 (Supplementary Table S1). *SEMA4A* encoded one class of semaphorin, which was often involved in immune responses and neurological diseases (Takegahara and Kumanogoh, 2010). For example, the SNP rs7702187 within *SEMA5A* (encoding another class of semphorins) was associated with PD (Clarimon et al., 2006). Other novel and significant gene loci (Supplementary Table S1) included *TRIM46*, *ASH1L*, *PBXIP1*, *RIT1*, and *PMF1-BGLAP*. The *RIT1* gene belongs to the Ras family related to neurodegenerative disorders (Qu et al., 2019).

### 3.3 Predictive ML models for PD developed using genetic data

Based on the GWAS results from the discovery (training) set, we further evaluated the capability of the top SNPs with the lowest *p*-values in predicting PD in an unseen test dataset. Within this context, GWAS served as a feature selection method for building our PD-predicting ML models. We experimented with various *p*-value thresholds (i.e., different numbers of top SNPs with the lowest *p*-values) and assessed model performance using an independent validation set. Among the tested thresholds, the *p*-value threshold of 1e-5, leaving us the top 37 SNPs (Supplementary Table S1), provided the best model performance (i.e., the highest AUC) in the validation set. This threshold was also commonly used for selecting SNPs in the development of PRS (Choi et al., 2020). Among these 37 SNPs, the three SNPs of rs76763715, rs1630500, and rs2049805 (Table 2; Supplementary Table S1) were published before as PD variants in other studies (Liu et al., 2011; Vacic et al., 2014; Davis et al., 2016). We further performed LD pruning using the ‘corr’ (correlation coefficient) method on the 37 SNPs and acquired 15 independent SNPs with a correlation coefficient threshold of 0.2. Table 2 lists these 15 independent SNPs and their nearest gene loci and variant type, minor-allele frequencies (MAFs), GWAS ranks*, p*-values, beta coefficients, and standard error (SE).

**TABLE 2:** Gene loci of potential influence on PD.

Variant	Gene: variant type	MAF	GWAS rank	GWAS *p*-value	GWAS beta	GWAS SE
rs76763715^a^	*GBA*: missense variant	0.016	1	3.03E-90	−4.113	0.204
rs1749409	*RIT1*: intron variant	0.091	7	2.55E-34	−1.824	0.149
rs1800247	*PMF1-BGLAP*: intron variant	0.212	12	8.47E-10	−0.898	0.146
i709741	None	0.107	15	3.39E-07	−0.777	0.152
rs11264300	*DCST1*: missense variant	0.366	17	8.31E-07	−0.836	0.170
rs4072037	*MUC1*: synonymous variant	0.472	22	1.30E-06	−1.065	0.220
rs75337321	*CACNA2D3*: intron variant	0.061	26	1.64E-06	−0.830	0.173
rs17377936	None	0.434	27	2.34E-06	0.682	0.145
rs58519469	*NTRK1*: intron variant	0.042	28	2.51E-06	−0.896	0.190
rs111408331	None	0.034	31	3.45E-06	−0.944	0.203
rs79372348	None	0.032	32	4.08E-06	−1.007	0.219
rs186852039	*GBA2*: intron variant	0.033	33	4.09E-06	−0.921	0.200
rs11772125	*AMZ1*: intron variant	0.069	34	4.30E-06	−0.799	0.174
rs11584630	*KCNN3*: intron variant	0.352	35	4.58E-06	−0.747	0.163
rs72792300	*ALK*: intron variant	0.015	37	7.46E-06	−1.178	0.263

^a^ Symbol next to variant ID indicates previously reported SNPs.

We used these 15 SNPs as the input features to train several ML models, including SVM, RF, LR, and ANN models. We tuned the hyperparameters for all four models based on their prediction performance in the validation set. In particular, the ANN model was trained using the stochastic gradient descent algorithm, with a batch size of 8, sigmoid activation functions, and a learning rate of 0.01. A three-layered ANN feed-forward network was used, consisting of one input layer, one hidden layer, and one output layer, while the hidden layer had four neurons. In Table 3, test set performance metrics are listed for all the developed ML models. As expected, when utilizing 15 randomly selected SNPs as the input features, the developed ANN model produced poor results, with an AUC of 0.50 and an F1-score of 0.49. When using the 15 independent SNPs identified by GWAS, the prediction performance of all the developed ML models (SVM, LR, RF, and ANN) greatly improved, with much higher AUCs and F1-scores. Among these, the ANN model performed the best overall, with a highest AUC of 0.74 and an F1-score of 0.64. We also derived a PRS from the 15 independent SNPs for each subject as the sum of their minor-allele SNP values, weighted by the log of their specific odds ratio from the GWAS analysis. The developed LR and ANN models from this one input feature of the PRS showed similar performance (the same AUC of 0.78, with an F1-score of 0.68 for ANN_PRS and 0.67 for LR_PRS), as expected. If compared to the best-performing ANN model using 15 independent SNPs, the ANN model using this one input feature of the PRS had better performance in PD prediction, with an improved AUC of 0.78 and a higher F1-score of 0.68; this is likely because the weights of these 15 SNPs used in calculating the PRS provided additional and useful information to help predict PD.

**TABLE 3:** Performance metrics of the genetic ML models for predicting PD.

ML model	AUC	Precision	Recall	F1-score
ANN_Random	0.50	0.49	0.50	0.49
SVM	0.67	0.58	0.70	0.60
LR	0.68	0.60	0.72	0.64
RF	0.68	0.57	0.65	0.59
ANN	0.74	0.69	0.61	0.64
ANN_PRS	0.78	0.65	0.72	0.68
LR_PRS	0.78	0.63	0.73	0.67
ANN_Combined	0.78	0.66	0.74	0.69

In addition to developing these genetic models, we further developed a combined ANN model using both genetic and demographic data. For this combined dataset, we had 12,070 subjects with 15 SNPs and 139 demographic variables as input features. We trained this combined ANN model similar to the genetic ANN model. Interestingly, with both genetic and demographic variables, the predictive performance of the ANN model was considerably increased to an AUC of 0.78 and an F1-score of 0.69 (Table 3).

The EG method was used to determine the top predictive SNPs in the ANN model, whereas the feature importance score was used in the RF model. Upon reviewing the top half (7 SNPs) identified by both methods, it was found that five of the top seven SNPs were shared between the two sets, suggesting a degree of agreement between the results generated by the two different methods applied to the two different ML models. The missense variant of rs76763715 located inside *GBA* was ranked first by RF FI and third by ANN ES, suggesting its high influence on PD prediction. This is consistent with the evidence that it is the most significant SNP with the lowest *p*-value in our GWAS analysis, and its association with PD has been validated in different studies. The intron variant rs1749409 in the *RIT1* gene, which was ranked seventh by GWAS *p*-values (2.55e-34), was ranked second by the RF FI and first by the ANN EG for its magnitude in influencing the PD prediction.

**FIGURE 3:**
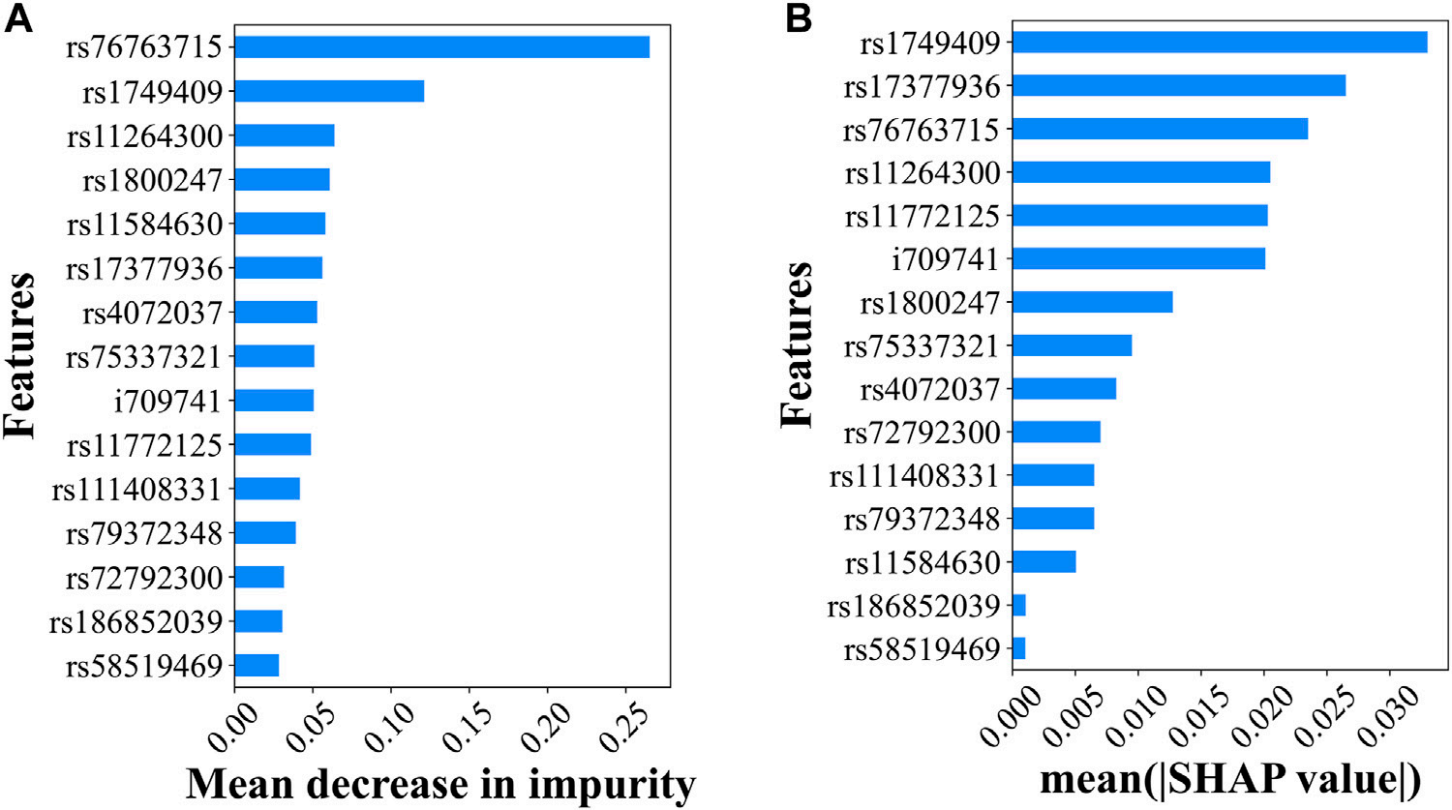
Top predictive genetic variants for PD. **(A)** The predictability of the 15 independent variants in the RF model explained by the feature importance scores and **(B)** the predictability of the 15 independent variants in the ANN model suggested by the SHAP values.

In addition to providing additional PD prediction evidence for some of the significant GWAS, SNPs, RF FI, and ANN EG also identified variants with decent predictive capability that were missed by GWAS (i.e., not reaching the significance level after the Bonferroni correction). The missense variant rs11264300 located in the *DCST1* gene was ranked 17th by GWAS (top 14 are significant) and third and fourth by RF FI and ANN EG, respectively (Figures 3A, B). Interestingly, a previous study identified an SNP in this *DCST1* gene as one of the most relevant PD polygenic risk score SNPs (Koch et al., 2021). These results suggested that this missense variant and the *DCST1* gene may have an important role in the development and progression of Parkinson’s disease. Similarly, RF FI identified rs11584630, an intron variant in the gene of *KCNN3*, as a very predictive variant (ranked fifth) for PD; interestingly, *KCNN3* was previously reported to be associated with PD pathogenesis (Simunovic et al., 2010).

### 3.4 Gene set enrichment analysis identified pathways associated with PD

**FIGURE 4:**
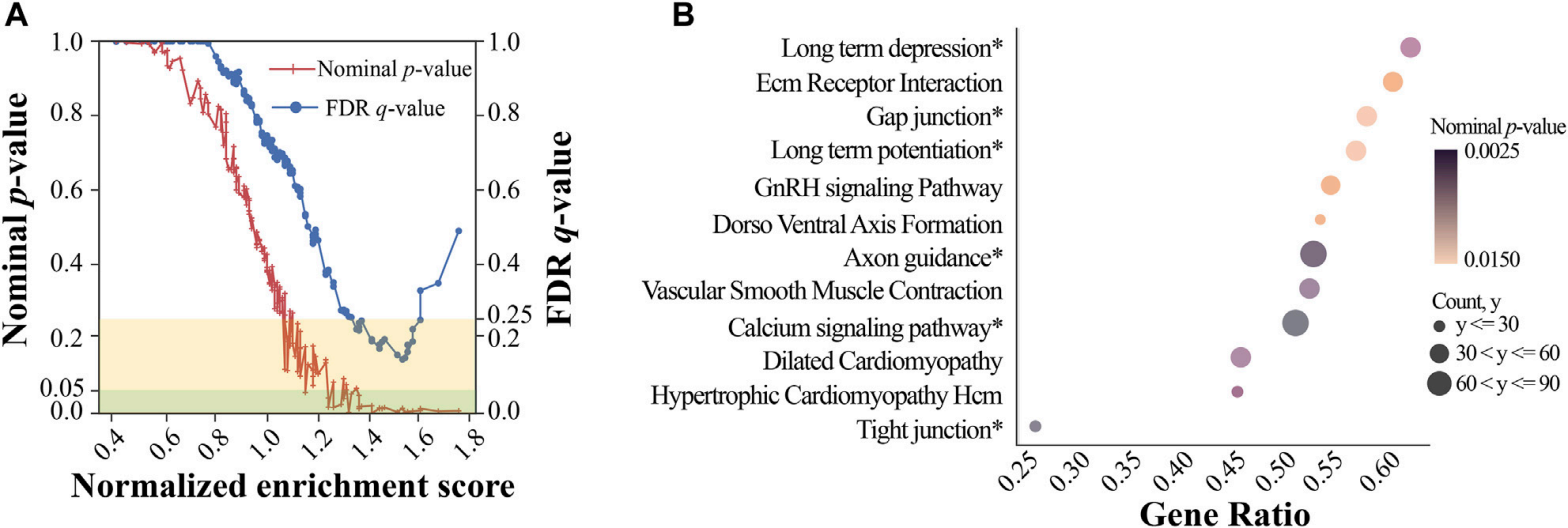
Gene set enrichment analysis. **(A)** Normalized enrichment score vs. significance plot. The red line represents the nominal *p*-value for gene sets, while the blue line represents the FDR q-value for gene sets. The yellow rectangle indicates the FDR levels within 0.25, and the green rectangle indicates the nominal p-value levels within 0.05; **(B)** a dot plot of top KEGG pathways. The size of each dot represents the number of core enrichment genes, while the color represents the nominal *p*-value. The names with the * symbol represent the pathways associated with PD, while others represent the potentially novel pathways associated with PD.

We used GWAS-based pathway analysis to further examine the potential PD pathways from the ranked gene list obtained by our GWAS analysis. GSEA was used to identify KEGG pathways significantly associated with PD. We used the minimum *p*-value among all SNPs near a gene to represent the significance of that gene (Wang et al., 2007). Initially, 166 gene sets (i.e., pathways) were identified by GSEA (Figure 4A) from which a total of 17 gene sets with relatively high NES were considered significant, reaching both the FDR (<0.25) and nominal *p*-value (<0.05) threshold (Supplementary Table S2). Figure 4B showed the top 12 (ranked by NES) significant pathways and their statistics, including the number of core enrichment genes, gene ratio, and nominal *p*-value. Gene ratio was calculated using the count of core enrichment genes divided by the count of pathway genes, whereas core enrichment genes were those that contribute most significantly (indicated by their *p*-values) to the observed enrichment of the gene set. Among the top 12 pathways, we identified six pathways whose functions were previously reported to be associated with PD (marked with a ‘*’ in Figure 4B): long-term depression, gap junction, long-term potentiation, axon guidance, calcium signaling pathway, and tight junction. One study found that corticostriatal long-term potentiation (LTP) and long-term depression (LTD) were altered in PD models (Calabresi et al., 2007). Schwab et al. (2014) showed that the gap junction protein Cx36 was upregulated in PD patients. Variations in axon guidance pathway genes were predictive of three PD outcomes (Lin et al., 2009). Calì et al. (2014) observed that calcium signaling was one of the earliest events in the pathogenesis of PD. The tight junction proteins occludin and ZO-1 were associated with the mouse model of Parkinson’s disease (Chen et al., 2008). The aforementioned literature supported the PD association for half of the top 12 GSEA pathways; this further strengthened the potential involvement of our top GWAS SNPs (or gene loci) in PD. In addition to identifying the aforementioned six pathways whose associations with PD were previously reported, our GSEA also identified novel pathways potentially associated with PD. Interestingly, three out of the six novel pathways we identified for PD, including the functions of the vascular smooth muscle (VSM) contraction, extracellular matrix (ECM) receptor interaction pathways, and gonadotropin-releasing hormone (GnRH) signaling pathway, although there is no strong evidence yet in the literature for their involvement in PD, were reported to be linked with other neural diseases such as Alzheimer’s disease (AD). This helped add support to the validity of our findings. For instance, the dysfunction of VSM cells (whose activity and responsiveness determine the dynamics of VSM contraction) was found to contribute to AD development by promoting neuroinflammation and Tau hyperphosphorylation (Aguilar-Pineda et al., 2021). Similarly, significant changes in ECM components occur during the early stages of AD (Anwar et al., 2022). Furthermore, increased mRNA levels of GnRH and its receptor were observed in plaque-bearing AD transgenic mice (Nuruddin et al., 2014). These pathways may serve as common pathways involved in different types of neural diseases, such as AD and PD.

## 4 Discussion

In this paper, we utilized a large collection of demographic and clinical variables together with the corresponding genomic data from the Fox Insight online study. We identified both novel and well-known demographic and genomic factors via correlation and GWAS analyses. From the top demographic and genomic factors, we further developed and compared a variety of ML models for predicting PD using demographic features alone, genomic features alone, and combined features considering both demographic and genomic factors. To understand the importance and predictability of the demographic and genomic factors, we performed EG analysis for the ANN demographic and genetic model as well as acquired feature importance scores from the RF demographic and genetic models. These input feature analyses, not yet adopted much by the PD domain, allowed us to interpret the ML models and identify the most predictive demographic and genomic factors for PD. Finally, we applied GSEA analysis based on our GWAS results and found both novel and previously reported PD pathways.

In the relatively large demographic dataset, both RF and ANN models did well with the same AUC of 0.89 and similar F1-scores of 0.79 and 0.80, respectively. The large overlapping of the top 14 demographic variables ranked by RF and ANN using two different feature analysis methods (RF feature importance and ES) strongly suggested the robustness of the models as well as the importance of these top demographic variables in PD prediction. As another line of evidence, most of these top demographic variables were reported previously for their association with PD. In the relatively small genetic dataset, the ANN model performed the best, and it also revealed the influence of each SNP feature on PD prediction; when including additional demographic features into the ANN model, the AUC and F1-scores were further increased to 0.78 and 0.69, respectively. The top predictive demographic and genetic features, together with the developed ML models, can potentially be used in the clinical setting to predict the PD risk before its onset for early intervention.

In this study, we performed rigorous experimentation to avoid potential overfitting and fair evaluation/analysis of the ML models as follows: 1) we strictly tuned hyperparameters for all the ML models based on their performance on a separate validation set and further evaluated the performance of the final model on an unseen test set; 2) we performed random bootstrapping for the control (non-PD) samples to get a more balanced dataset for the training set only to avoid potential information leakage and overfitting in the validation or test set; 3) in addition to looking at the evaluation metric of AUC, we also examined the precision, recall, and F1-score of all the ML models for a more comprehensive and less biased evaluation; and 4) we compared and developed different ML models for PD prediction, where we used two different methods (RF feature importance and EG) to analyze and understand the feature importance from two different models (RF and ANN). Despite these rigorous experimental designs, it would be ideal if we could obtain additional data and further validate these ML models on an independent study.

Through correlation analysis, GWAS, and feature importance analysis, we identified both novel and well-known demographic and genetic factors related to PD. For example, in our GWAS analysis, we identified well-known variants in the *GBA* gene, which encodes the glucocerebrosidase enzyme implicated in Gaucher’s disease, a lysosomal storage disorder. It had been established that lysosomal dysfunction, associated with *GBA* gene mutations, was linked to neurodegeneration and, particularly, to Parkinson’s disease (Navarro-Romero et al., 2020). Our findings reinforced the importance of the *GBA* gene lysosomal pathways in the pathophysiology of PD. Other than identifying well-known variants within *GBA*, our GWAS analysis also identified several novel and significant variants and gene loci; among these, three novel intron variants in *LMNA* (p-values smaller than 4.0e-21) and one novel missense variant in *SEMA4A* (p-value = 1.11e-26) with very small *p*-values are particularly interesting, since *LMNA* and semphorins were reported to be associated with PD by other studies. The gene *SEMA4A* had previously been linked to Th17 cell-mediated neuroinflammation (Koda et al., 2020). Given that neuroinflammation is a well-recognized component of PD pathology, our findings suggested a potential role of *SEMA4A* in the progression of PD, potentially via modulating neuroinflammatory processes. Our feature importance analysis from the PD-predicting ANN and RF models provided another set of evidence to show the capability of the variants in predicting PD. These analyses highlighted some of the significant variants identified by GWAS, such as the well-known missense variant of rs76763715 located inside *GBA* and the intron variant rs1749409 in the *RIT1* gene, both of which were ranked within the top three most-predicting variants by both RF FI and ANN EG; these ML feature importance analyses also helped identify rs11264300, a missense variant in the gene of *DCST1*, and, rs11584630, an intron variant in the gene of *KCNN3*—although these variants did not reach the GWAS significance, their corresponding genes were reported to be associated with PD by other studies (Simunovic et al., 2010; Koch et al., 2021). Overall, this coupling of ML approaches with the GWAS analysis is beneficial in validating the significance of GWAS-identified PD variants with additional PD prediction evidence and identifying potential PD variants that could have been missed by GWAS due to limited power.

## 5 Conclusion

In summary, by performing GWAS analysis coupled with ML approaches, we identified impactful demographic and genomic factors as well as developed ML models that may help predict PD. The new loci identified from GWAS or ML input feature importance analysis warranted further investigation.

## Data Availability

The data analyzed in this study were obtained from the Fox Insight study via the Fox Insight Data Exploration Network (Fox DEN; https://foxden.michaeljfox.org/insight/explore/insight.jsp), and the following licenses/restrictions were applied: qualified researchers may apply for access to Fox Insight datasets. Requests to access these datasets should be directed to Fox DEN: https://foxden.michaeljfox.org/insight/register/genetic.
